# Composition of Different Herbal Extracts and Their Impact on Initial Bacterial Colonization on Enamel In Situ

**DOI:** 10.3390/plants15132101

**Published:** 2026-07-07

**Authors:** Theresa Schneider, Isabelle Kölling-Speer, Sarah Hellmann, Cindy Scheunemann, Karl Speer, Christian Hannig, Matthias Hannig, Jasmin Flemming

**Affiliations:** 1 Department of Prosthodontics, Faculty of Medicine and University Hospital Carl Gustav Carus, TUD Dresden University of Technology, Fetscherstraße 74, 01309 Dresden, Germany; 2 Department of Operative Dentistry, Periodontolgy and Pediatric Dentistry, Faculty of Medicine and University Hospital Carl Gustav Carus, TUD Dresden University of Technology, Fetscherstraße 74, 01309 Dresden, Germany; 3 Special Food Chemistry and Food Production, TUD Dresden University of Technology, Bergstraße 66, D-01069 Dresden, Germany; 4 Clinic of Operative Dentistry, Periodontology and Preventive Dentistry, University Hospital, Saarland University, Building 73, D-66421 Homburg, Germany

**Keywords:** polyphenols, blackcurrant leaves, *Ribis nigri* folium, oak bark, *Quercus* cortex, horse chestnut leaves, *Hippocastani* folium, sweet chestnut leaves, *Castaneae* folium

## Abstract

Foods rich in polyphenols are known to promote oral health by modifying the enamel pellicle. In doing so, they reduce bacterial adhesion, biofilm maturation, and erosion. The goal of this study was to screen local herbal drugs available in Central Europe for their potential suitability as part of a diet promoting oral health by targeting the initial stages of biofilm formation. To achieve this, an in situ study was conducted to evaluate the effects of the four polyphenol-rich herbal extracts of blackcurrant leaves, oak bark, horse chestnut leaves, and sweet chestnut leaves on early bacterial adhesion and biofilm formation on tooth enamel over an 8 h period. This research aimed to identify natural remedies that could support oral hygiene by targeting the initial stages of biofilm formation. Study Design and Experimental Procedures: Aqueous extracts were prepared by ultrasonic extraction. Eight human subjects wore bovine enamel slabs intraorally for 8 h. After 1 min of pellicle formation, the subjects rinsed with 8 mL of the extracts for 10 min, followed by intraoral exposure without food. An 8 h-exposure without rinse served as the negative control; 0.2% chlorhexidine gluconate (CHX) served as the positive control. After 8 h, bacterial adhesion and biofilm matrix formation on the enamel slabs were quantified ex vivo using DAPI/Concanavalin A staining and fluorescence microscopy. The LIVE/DEAD™ BacLight™ assay was used to assess bacterial viability. Statistical analysis was performed by the Mann–Whitney U test and Kruskal–Wallis test (*p* < 0.05), as well as the Bonferroni–Holm correction (*p* < 0.01). Results and Conclusions: The screened herbal drugs did not demonstrate a statistically significant impact on the number of adherent bacteria, suggesting that their mode of action may not directly interfere with bacterial adhesion mechanisms. However, all four extracts exhibited consistent trends toward reduced glucan formation and decreased bacterial viability. The observed inhibition of glucan formation indicates that these drugs may potentially target the enzymatic pathways responsible for polysaccharide synthesis. By disrupting glucan production, the structural integrity of the biofilm matrix might be compromised, which indirectly affects bacterial survival within the biofilm environment.

## 1. Introduction

The increasing global prevalence of dental diseases such as caries, periodontitis, and erosion calls for improved oral health interventions, particularly those focused on prevention and a health-promoting diet. At the same time, a growing number of patients are demanding sustainable, natural, and locally sourced alternatives to conventional anti-caries agents. The process of biofilm formation on tooth surfaces plays a defining role in the development of these pathologies. Initial bioadhesion is a critical step in microbial colonization on dental enamel. The tooth surface is known to be the only non-shedding surface in the human body. Its lack of regenerative capabilities makes it particularly vulnerable to bacterial colonization. Central to this process is the formation of the acquired enamel pellicle, which is a protein-rich film that forms immediately upon exposure of the tooth surface to saliva. The acquired pellicle serves as an essential mediator of microbial adhesion, facilitating the early stages of biofilm development and influencing subsequent bacterial interactions. Despite its protective function, the acquired enamel pellicle can also serve as a target for intervention. Secondary plant metabolites, particularly polyphenolic compounds, have received attention due to their potential to modulate the acquired enamel pellicle properties and reduce bacterial adhesion. This offers a promising approach for the prevention of dental diseases.

Polyphenols are reported to denature and crosslink pellicle proteins, a process known as the tanning effect [1]. Through these tanning effects on bacterial enzymes, glucan formation can be inhibited, which plays an important part in bacterial adherence [1,2]. Moreover, polyphenols can interact with bacterial membranes and complex metal ions, leading to antibacterial effects [3].

Polyphenols, abundant in many plants, are known for their antioxidant, antibacterial, and anti-inflammatory properties. These compounds, such as flavonoids, tannins, and phenolic acids, play a vital role in the plant’s defense mechanisms and exhibit a variety of biological activities that may extend to oral health. Schestakov and Hannig observed both strong anti-adherent and antibacterial effects on enamel in situ after rinsing with tannic acid, but the effects declined over time. Transmission electron micrographs of salivary samples after rinsing with tannic acid indicated that the aggregation of proteins and bacteria might explain the anti-adhesion effects of tannic acid [4].

The goal of this study was to screen polyphenol-rich herbal drugs available in Central Europe for their potential in reducing the initial stages of biofilm formation. Based on the literature, four polyphenol-rich herbal drugs (blackcurrant leaves, oak bark, horse chestnut leaves, and sweet chestnut) were selected, as they appear suitable for promoting oral health without impairing the physiological function of the oral biofilm. The selection criteria included taste, high content of polyphenols, especially tannins, and pH level, as a lower pH level enhances the binding of polyphenols to oral structures.

A chemical analysis of their herbal extracts was performed and combined with a well-established in situ study mode to evaluate the effects of four herbal extracts—blackcurrant leaves, oak bark, horse chestnut leaves, and sweet chestnut leaves—on early bacterial adhesion and biofilm formation on tooth enamel over an 8 h period. By focusing on the anti-adherent properties of these plant compounds, we aim to determine their efficacy in modulating the acquired enamel pellicle’s protective capabilities and preventing the early stages of biofilm formation. This study addresses how the four selected polyphenol-containing herbal extracts influence initial bacterial adhesion to the enamel surface in terms of the quantitative and qualitative composition of adherent bacteria and the resulting biofilm matrix.

Blackcurrant leaves are described in the literature as containing 0.5–1.2% flavonoids, in particular glycosides of kaempferol and quercetin, such as astragalin, isoquercitrin, and rutin. They also contain flavonoid glycosides of myricetin and isorhamnetin. In addition, approximately 0.4% oligomeric proanthocyanidins, a precursor of tannins, have been detected. The leaves of blackcurrants contain between 0.1 and 0.27% ascorbic acid, as well as traces of essential oils and various phenolic acids [5,6,7,8,9,10,11,12,13]. The highest polyphenol content is found in the leaves of the plant, which is significantly higher than in the flowers or berries [10]. The consumption of blackcurrants before smoking was able to reverse the reduction in the salivary flow rate and the secretion rate of secretory immunoglobulin A in saliva induced by smoking in situ [14]. This suggests that the consumption of blackcurrants may boost oral clearance. Ikuta et al. demonstrated the antiviral and antibacterial effects of blackcurrant leaf extract against oral and nasopharyngeal viruses and bacteria in vitro, although their study showed no antibacterial effects against *Streptococcus mutans* [15]. Weber et al. demonstrated an anti-erosive effect after 10 min (minutes) of rinsing with blackcurrant leaf extract, which was further enhanced by combining it with oregano extract [16].

According to the literature, tannins dominate oak bark, accounting for 12 to 16% of its content. In addition to ellagitannins, flavonolic ellagitannins, and a procyanidinoellagitannin, condensed non-hydrolyzable catechin tannins consisting of catechin, epicatechin, and gallocatechin have been identified; furthermore, 5-desoxyinositol (quercitol) and triterpenes have also been found [12,13,17,18,19,20,21]. The tannin-containing drug has astringent, anti-inflammatory, antiviral, and anthelmintic effects [12]. Oak bark extract exhibits weak antibacterial and pronounced anti-quorum sensing effects [22]. The astringent effect of tannins causes soluble proteins to be converted into insoluble proteins, thereby inhibiting the secretion of superficial glands in the skin and mucous membranes [12].

Horse chestnut leaves are rich in flavonols such as quercetin and kaempferol, as well as flavonol glycosides such as quercitrin, and contain traces of aescin. [12,13,23,24,25,26,27,28]. Extracts of chestnut leaves showed no inhibitory effect on *Bacillus cereus*, *Staphylococcus epidermidis*, *Proteus vulgaris*, and *S. pneumonia* in vitro, but did show a weak inhibitory effect on the growth of Listeria monocytogenes, Klebsiella pneumoniae, and Staphylococcus aureus. No antifungal activity could be detected [29]. In a canine model, horse chestnut leaves extract showed an inhibitory effect on host-derived matrix metalloproteinases (MMPs). MMPs are the most important proteinases associated with the destruction and remodeling of periodontal tissue [23].

According to the literature, sweet chestnut leaves contain 6 to 8% tannins from the ellagitannin group, as well as flavonoids, particularly rutin, quercetin, and myricetin derivatives, triterpenes, 0.2% vitamin C, and gallic acid [12,13,30,31,32,33,34,35]. Quave et al. demonstrated that chestnut leaf extract inhibits quorum sensing in *Staphylococcus aureus* without inhibiting the growth of skin commensals or causing cytotoxicity in human keratinocytes in vitro [36]. Due to its high content of tannins and flavonoids, chestnut leaf extract has strong antioxidant and radical scavenging properties, as well as anti-inflammatory activities due to the polar lipids it contains [37]. An in vitro study by Basile et al. demonstrated that the extract showed a pronounced antibacterial effect against seven of the eight Gram-positive and Gram-negative bacterial strains used, with *Enterobacter aerogenes* and *Staphylococcus aureus* already showing sensitivity at lower concentrations. Basile et al. identified rutin, hesperidin, quercetin, apigenin, morin, naringin, galangin, and kaempferol as active fractions of the sweet chestnut extract used. Testing of the individual flavonoids identified as standard solutions against the same bacterial strains showed the highest antibacterial activity for quercetin, rutin, and apigenin [38].

This has led to an interest in exploring these herbal extracts as natural agents in oral hygiene regimens or as functional foods focusing on oral health. In situ studies have shown that some polyphenolic herbal extracts have the potential to modulate pellicle formation [1], thereby enhancing its protective function and reducing bacterial adhesion [3]. Given the complex variability in polyphenol composition among plant sources and the heterogeneous nature of bacterial communities within dental biofilms, screening plants for their application in oral health may unveil novel, sustainable agents capable of targeting critical mechanisms in biofilm development, such as bacterial adhesion, glucan synthesis, and microbial viability.

## 2. Study Design and Experimental Procedures

### 2.1. Subjects

A total of eight subjects (four female, four male, aged 22–34 years) participated in the present in situ study. An experienced dentist carried out visual oral examination. The study was conducted at the department of operative dentistry at the Medical Faculty Carl Gustav Carus, Dresden, Germany. The ethics committee of the TUD Dresden University of Technology approved the study design (Vote: EK 275092013).

The inclusion criteria were as follows:

- Competent adults;
- Good oral hygiene;
- Written informed consent provided;
- Clinically healthy oral status confirmed by visual examination;
- Caries-inactive, with no signs of periodontal disease or carious lesions in the last two years.

The exclusion criteria were as follows:

- Any medical condition or relevant medical history;
- Current medication use;
- Smoking;
- Presence of oral pathology detected during clinical examination.

### 2.2. Specimens and In Situ Positioning

In the present study, bovine enamel slabs were selected as specimens. Bovine enamel is an established and available source as a human enamel substitute in dental research [4,39]. Due to the great similarities in chemical composition and physical properties of bovine and human enamel, bovine enamel samples are suitable as specimens for the analysis and evaluation of microbial adhesion [40].

A total of 192 cylindrical enamel slabs (5 mm in diameter, 19.63 mm^2^ surface area, and 1.5 mm in height) were prepared from the labial surfaces of bovine incisors using a diamond-coated trephine drill. The teeth were a by-product of meat production from BSE-negative two-year-old cattle that had been examined by a veterinarian and approved for human consumption. The teeth were extracted from freshly slaughtered cattle, cleaned and stored in thymol solution at 4 °C until further processing. Enamel slabs with structural alterations like cracks were excluded from the study.

The enamel surfaces were polished by wet grinding with abrasive paper (320, 1200 and 4000 grit). The specimens were then treated and disinfected according to the following protocol:

A 3 min ultrasonic bath in 3% NaOCl was followed by two consecutive 5 min ultrasonic baths in distilled water, a 5 min ultrasonic bath in 70% ethanol, and storage in distilled water for at least 24 h before use for rehydration purposes.

An individual splint was made for each subject with stainless-steel wire and methyl methacrylate (Forestacryl^®^; Forestadent Bernhard Förster GmbH, Pforzheim, Germany) for in situ positioning of the specimens. Four enamel slabs were mounted into the splints using a low-viscosity silicone impression material (Provil^®^ novo light, Kulzer GmbH, Hanau, Germany) and positioned in the vestibular region corresponding to the second premolars and first molars of the upper jaw, with two slabs on each side.

### 2.3. Medicinal Plant Materials

Four herbal drugs (Table 1) were selected based on their polyphenol profiles and proven range of applications as potentially anti-adherent and biofilm-inhibiting agents. They originate from the leaves or bark as outer parts of plants native to Central Europe. All four herbal drugs have high polyphenol content in common, although they have individual flavonoid and tannin profiles, as well as pH values.

### 2.4. Extraction and Analysis of the Herbal Drugs

First, 20 g of the individual herbal drug was finely ground using an IKA Tube Mill 100 control with an MT 40 milling tube (IKA-Werke, Staufen, Germany) for 20 s at 15,000 rpm.

For extraction, 0.8 g of freshly prepared powder was mixed with 10 mL of double-distilled water in a 15 mL PTFE tube (VWR, Radnor, PA, USA) and exposed to an ultrasonic bath at 20 °C for 30 min. After 10 min centrifugation at 10,000 rpm, the supernatant was transferred to a 25 mL volumetric flask. This process was repeated with another 10 mL of double-distilled water. The united extracts in the 25 mL volumetric flask were filled up to the mark with double-distilled water. A ratio of 0.8 g of tea to 25 mL of water was chosen to achieve a flavor with an acceptable level of astringency, thereby ensuring good compliance among the participants during the 10 min oral rinse.

Each drug preparation was carried out four times, ultimately achieving 100 mL of extract.

Then, 10 mL of the extract was filtered using a 0.2 µm membrane filter (Chromafil GF/RC-20–25, 25 mm regenerated cellulose, Macherey & Nagel, Düren, Germany) directly into a 15 mL tube for oral application; for HPLC (High-Performance Liquid Chromatography with a Diode Array Detector (DAD) and Mass Spectrometer (MS)) analyses, aliquots of the extracts were filtered into a 1.5 mL vial. The extracts were stored in these tubes at −20 °C until use. Chemical analyses of the freshly prepared and frozen extracts showed no significant differences. 

### 2.5. HPLC/DAD-MS

The Chromaster HPLC system (Hitachi, Tokyo, Japan) with a VWR diode array detector (VWR-Avantor, Radnor Township, PA, USA), in combination with the TSQ Quantum MS (Thermo Fisher Scientific GmbH, Dreieich-Buchschlag, Germany), was used for the HPLC/DAD-MS analysis. The separation was achieved by applying a Nucleodur Sphinx RP-C18 HPLC column, 5 µm, 250 × 3 mm (Macherey & Nagel, Düren, Germany) at 22 °C with an acetonitrile–water (with 0.5% acetic acid, MS grade) gradient and a flow rate of 0.25 mL/min. The gradient profile was 0–35 min from 8 to 30% acetonitrile, from 35 to 43 min from 30 to 90%, then hold for 15 min. The injection volume was 5 µL and the DAD wavelength range was 200 to 600 nm. The chromatograms of wavelengths 272 nm and 354 nm were extracted to visualize the group of tannins and the group of flavonols, as the relevant polyphenols exhibit maximum absorption in these two ranges. The identification of individual components was carried out with MS in negative ESI-Mode using standard substances or by analyzing extracts from other plants with known composition. Quantification: Since standards are often not available or could not be purchased, phenolic compounds were quantified in regard to compounds belonging to the same substance group and showing a comparable UV maximum: gallotannins as gallic acid (272 nm; G), phenolic acids as 5-CQA (324 nm; C), flavonol glycosides as rutin (354; R), and ellagitannins as ellagic acid (272 nm; EA) or as vescalagin (224 nm, V).

### 2.6. Study Protocol

**Figure 1:**
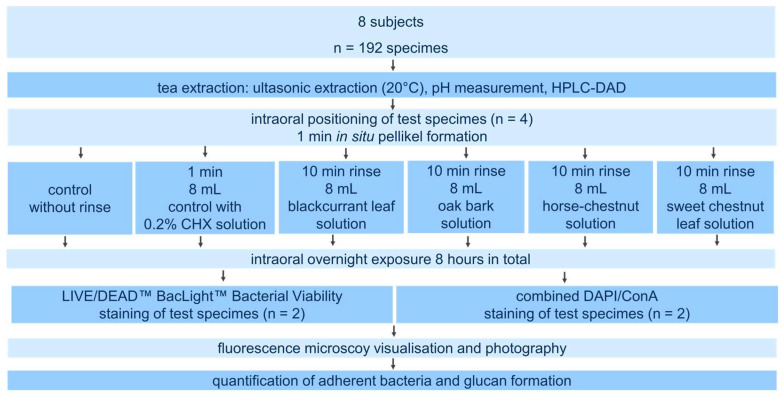
Flowchart of the study protocol.

The experimental setup is illustrated in Figure 1. All study participants took part in 6 trial runs on different nights, with a washout period of 2 days between each run. All subjects were instructed to brush their teeth with a manual toothbrush (Soft Protection, Dontodent, Karlsruhe, Deutschland, Germany) and tap water without toothpaste before going to bed and were only permitted to consume tap water afterwards.

For initial pellicle formation, the participants carried their splint with 2 specimenson each side for 1 min. Thereafter, they rinsed with the test solutions, either for 10 min with one of the tea extracts or with CHX for 1 min. A 10 min rinse with the plant extracts was chosen to simulate the consumption of a cup of tea in several sips. For further biofilm formation, participants continued to wear their splints for an additional 7 h (hours) and 49 min following the rinse with the plant extracts and 7 h and 58 min following the rinse with CHX, respectively. As a control group, all participants wore their splint for a period of 8 h without rinsing with any solution. Samples were stored in a moist tissue at 4 °C for transport for a maximum of 60 min until the microscopic analysis.

### 2.7. Visualization of Bacterial Adhesion, Viability, and Glucan Formation

A combination of 4′,6-diamidino-2-phenylindole (DAPI) and Alexa Fluor 574-conjugated Concanavalin A (Con A), hereinafter referred to as DAPI and Con A, is a well-established method to visualize adherent biofilm on enamel specimens [4,39,41]. DAPI binds to double-stranded bacterial DNA and stains adherent bacteria, while Con A binds specifically to α-mannopyranosyl and α-glucopyranosyl residues of glucans as characteristic representatives of the extracellular polysaccharides in the oral biofilm matrix.

To evaluate the viability status of the adherent bacteria, LIVE/DEAD™ BacLight™ staining (Invitrogen, Molecular probes, Darmstadt, Germany) was performed. The combination of the green-fluorescent dye SYTO^®^ 9 (component A, 1.67 mM/propidium iodide, 1.67 mM, 300 µL DMSO), which can penetrate both bacteria with either intact or damaged membranes, with the red-fluorescent dye Propidium iodide (component B, 1.67 mM/propidium iodide, 18.3 mM, 300 µL DMSO), which can only penetrate damaged membranes and displaces the SYTO^®^ 9 dye, is used for fluorescence-based vital staining of bacteria, enabling differentiation between vital and dead bacteria based on their membrane integrity only. The LIVE/DEAD™ BacLight™ assay also allowed the calculation of cell viability, defined as the percentage of vital bacteria.

Epifluorescence microscopic analysis was performed at ×1000 magnification (Axioskop II; ZEISS) using the following light filters: DAPI (BP 381-399, FT 416, LP 430-490); Texas Red (BP 542-576, FT 585, LP 595-664); and FITC (BP 458-493, FT 499, LP 509-552) (Carl Zeiss Microscopy GmbH, Oberkochen, Germany). The number of bacterial cells was counted manually in 10 randomly selected 100 µm × 100 µm microscopic grid squares on the enamel slabs, and the average density of adherent bacteria per cm^2^ was calculated from these counts.

The glucan structures were evaluated using a well-established scoring system (Table 2) [39,41,42].

### 2.8. Statistics

Statistical analysis was performed using SPSS 28.0 (IBM, Ehningen, Germany) and the graphical representation was created using Origin 2021b (OriginLab Corporation, Northampton, MA, USA). The data were analyzed using Kruskal–Wallis and Mann–Whitney-U tests as the values were not normally distributed, followed by Bonferroni correction. The hypothesis was that the distribution of values is consistent across all tested groups. The significance level was set at *p* < 0.05 after Bonferroni correction. Should the power exceed 0.8, the result of the statistical test was considered reliable.

## 3. Results

### 3.1. Characteristics of the Herbal Extracts

The pH values (Table 3) of the aqueous extracts of blackcurrant leaves, horse chestnut leaves, sweet chestnut leaves and oak bark differed considerably.

**Figure 2:**
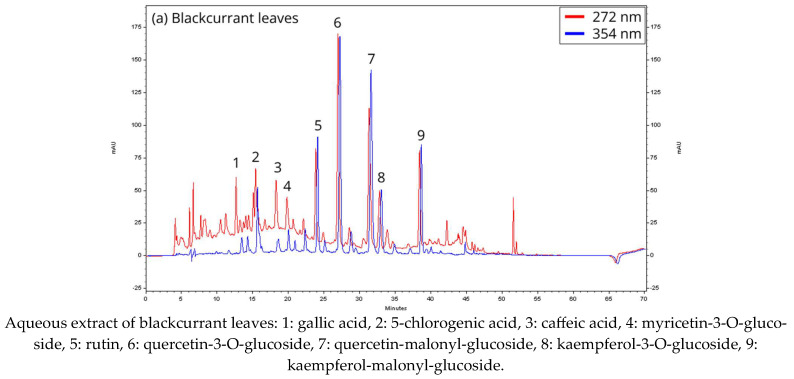

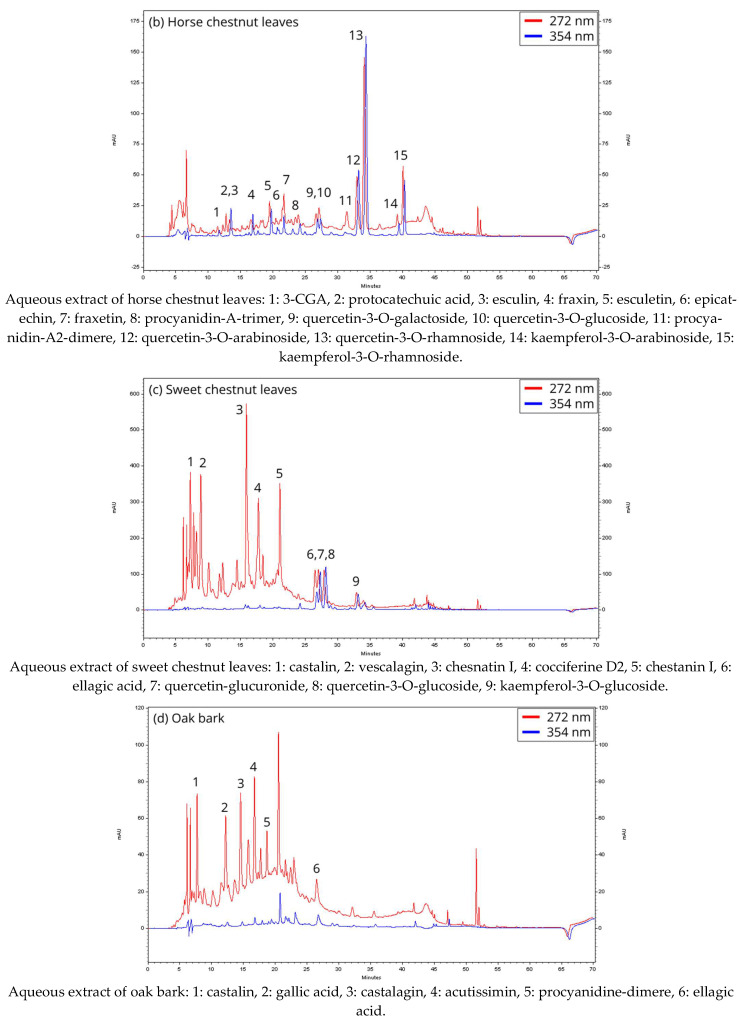
(**a**–**d**) HPLC chromatograms of the aqueous extracts of the four herbal drugs.

The HPLC-DAD chromatograms, acquired with the same gradient and depicted at wavelengths of 272 nm (red line, for tannins) and 354 nm (blue line, for flavonoids), are presented in Figure 2a–d.

The chromatograms of the four matrices reveal differences in their composition and in the concentration of the individual components. Blackcurrant leaves contain a variety of phenolic acids and, with the glycosides—mainly the glucosides—of myricetin, quercetin, kaempferol, and isorhamnetin, also a variety of flavonol compounds. Malonylglycosides are particularly noteworthy. The content of condensed tannins, on the other hand, is extremely low.

The horse chestnut leaves contained fewer phenolic acids, and as flavonol components, the arabinosides and rhamnosides of kaempferol and quercetin, rather than the glucosides, were the main components. Additionally, condensed tannins and, as a special feature, derivatives of the coumarin components esculetin and fraxetin were detected.

The sweet chestnut leaves contained hardly any phenolic acids but several flavonol glycosides of quercetin, kaempferol, and isorhamnetin in almost the same amounts as the blackcurrant and horse chestnut leaves, with the glucuronides being worth mentioning. The main components, however, are the ellagitannins with derivatives of vescalagin and castalagin.

The ellagitannins vescalagin and castalagin were also components of the oak bark analyzed. Above all, several complex flavo-tannins such as acutissimin and mongolicanin were detected, whereas condensed tannins, phenolic acids, and flavonols were present only in minor amounts. Detailed information of the individual components and their quantitative data for the four herbal drugs analyzed can be taken from the Supplementary Materials.

### 3.2. Biofilm Characteristics

**Figure 3:**
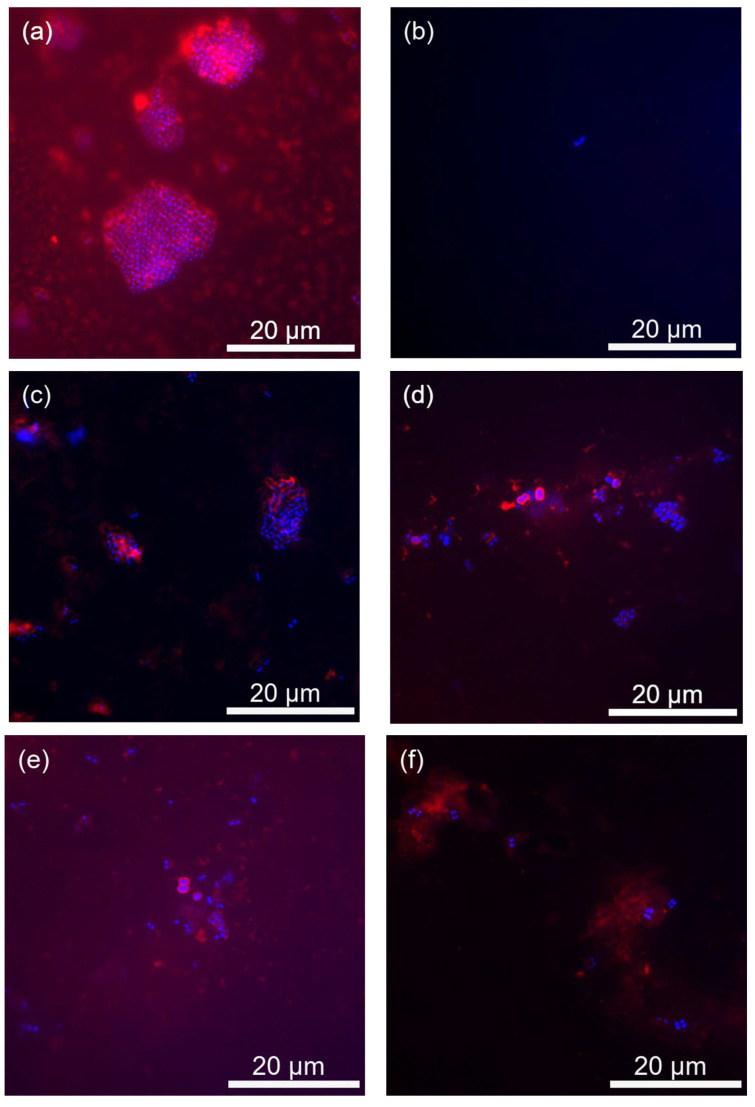
Representative fields of the enamel surface after 8 h oral exposition following no rinse (**a**), CHX (**b**), blackcurrant leaves (**c**), oak bark (**d**), horse chestnut leaves (**e**) and sweet chestnut leaves (**f**). Adherent bacterial cells are highlighted in blue, while surrounding bacterial glucan agglomerates are labeled in red.

Figure 3 shows representative regions of the enamel surface after 8 h of oral exposition and staining. The DAPI dye was used to visualize the total number of adherent bacteria regardless of their vitality status. Simultaneous staining with Con A was used to visualize the glucan structures as a component of the extracellular polysaccharide matrix. The epifluorescence microscopic images after combining DAPI and Con A staining provide not only the total number of adherent bacteria but also information about their arrangement within the biofilm and the density of the glucan structures.

**Figure 4:**
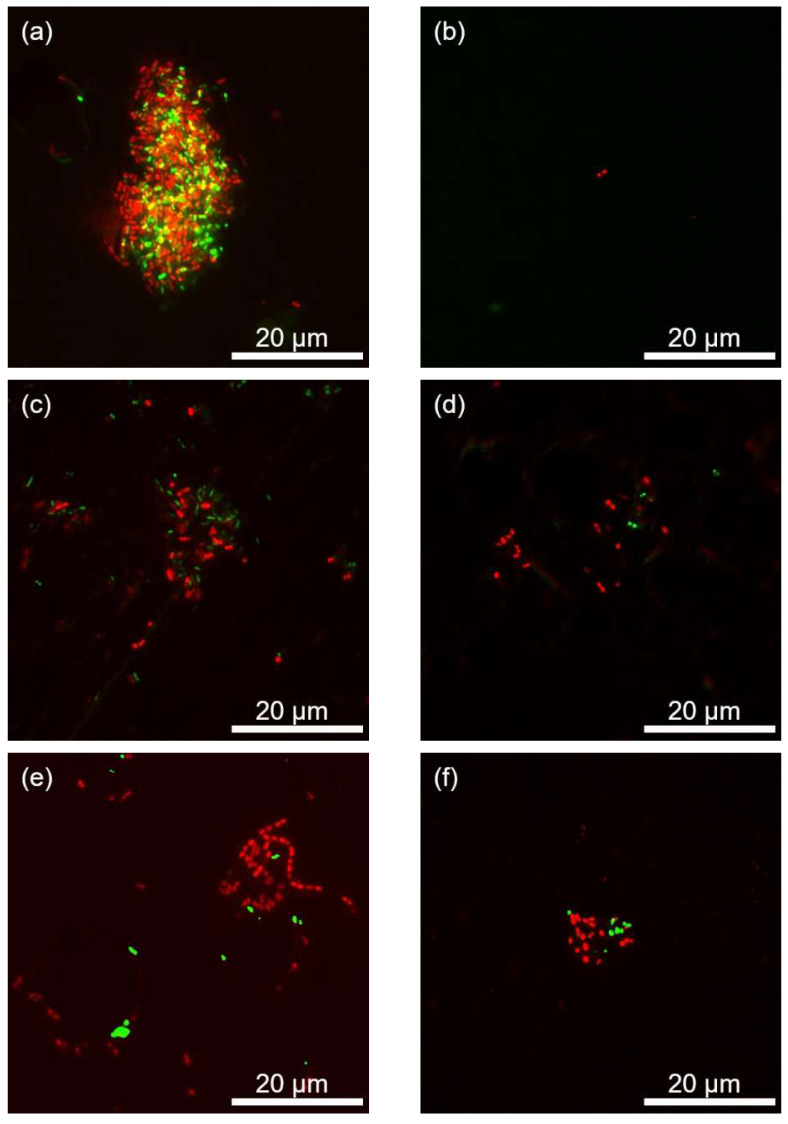
Representative fields of the enamel surface after 8 h oral exposition following no rinse (**a**), CHX (**b**), blackcurrant leaves (**c**), oak bark (**d**), horse chestnut leaves (**e**) and sweet chestnut leaves (**f**). Vital adherent bacterial cells are labeled in green, while dead bacterial cells are labeled in red.

The epifluorescence-based LIVE/DEAD™ BacLight™ staining procedure was employed to visualize adherent bacteria and to differentiate between vital and dead cells, thereby allowing the assessment of bacterial viability ratios (Figure 4).

#### 3.2.1. Control

**Figure 5:**
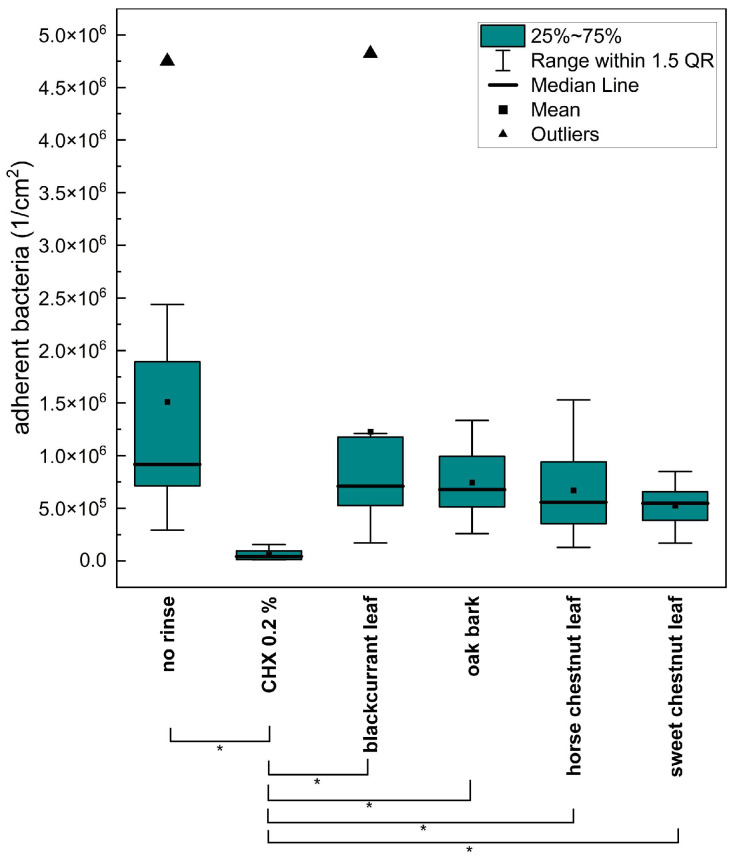
Density of adherent bacteria on enamel after 8 h of oral exposure following no rinse, CHX rinse, or 10 min rinsing with the four plant extracts. Asterisks indicate significant differences (Mann–Whitney U test with Bonferroni–Holm correction, *p* < 0.05).

The highest bacterial density of adherent bacteria occurred in the negative control without rinse, which can be regarded as the baseline value. An extensive single-layer bacterial colonization could be detected on the enamel slabs of most subjects, as shown in Figure 3a. The microorganisms were primarily coccoid bacteria in large agglomerates, smaller groups or chains. The specimens of the individual subjects were characterized by high inter-individual variability in the number of bacteria detected and their distribution, which is reflected in a strong scattering of the measured values. The statistical analysis illustrated in Figure 5 shows a comparative boxplot diagram of the bacterial colonization regardless of their vitality status. The most developed glucan film was found in the negative control sample. The majority of bacteria were embedded in an extracellular matrix of polysaccharides (Figure 3a). Both vital and dead bacteria were detected in comparable proportions (Figure 4a).

#### 3.2.2. 0.2% CHX

In the control group with 0.2% CHX rinse and 8 h of oral exposure, sparse bacterial colonization and no recognizable glucan rings on the test specimens were found (Figure 3b). A significant reduction in bacterial adherence and glucan score compared to the negative control was demonstrated, which characterizes the 0.2% CHX rinse as a suitable positive control. Rinsing with CHX also resulted in a pronounced reduction in both vital and dead bacteria (Figure 4b). Only a few predominantly dead cells were detectable. Desquamated epithelial cells were frequently observed.

#### 3.2.3. Blackcurrant Leaves

Specimens exposed to blackcurrant leaf extract displayed small groups and agglomerates of coccoid bacteria, as shown in Figure 3c. The glucan structures appeared as cloud-like formations, with individual glucan rings surrounding the adherent bacteria, some faint and others clearly visible. A shift in the live/dead ratio toward a dominance of dead bacteria was observed (Figure 4c).

#### 3.2.4. Oak Bark

The bacterial colonization pattern 8 h after rinsing with oak bark extract occurred predominantly in small groups or chains of streptococci, and rarely as large agglomerates (Figure 3d). The extracellular polysaccharides were mainly present as clear rings, and less frequently as weak rings or clouds. The LIVE/DEAD™ BacLight™ vital staining revealed mainly scattered dead bacteria, present singly or in small groups and chains. Dead bacteria predominated (Figure 4d).

#### 3.2.5. Horse Chestnut Leaves

Bacterial colonization on the enamel surface appeared as small groups, chains, and larger clusters of coccoid bacteria after rinsing with horse chestnut leaf extract (Figure 3e). The glucan structures were mostly visible as clear rings, and more rarely also as weak rings.

Rinsing with horse chestnut leaf extract yielded predominantly dead cells (Figure 4e).

#### 3.2.6. Sweet Chestnut Leaves

Bacteria were detected on the specimens in isolated cases, in small groups or strands, and in medium-sized clusters after rinsing with sweet chestnut leaves and 8 h of oral exposure (Figure 3f). Glucan structures mostly appeared as clear rings, rarely as faint rings or clouds, and were detectable around more than half of the adherent bacteria.

A trend has been noticed whereby both dead and, especially, vital bacteria were reduced relative to the negative control (Figure 4f).

### 3.3. Comparison of Individual Rinsing Solutions

**Figure 6:**
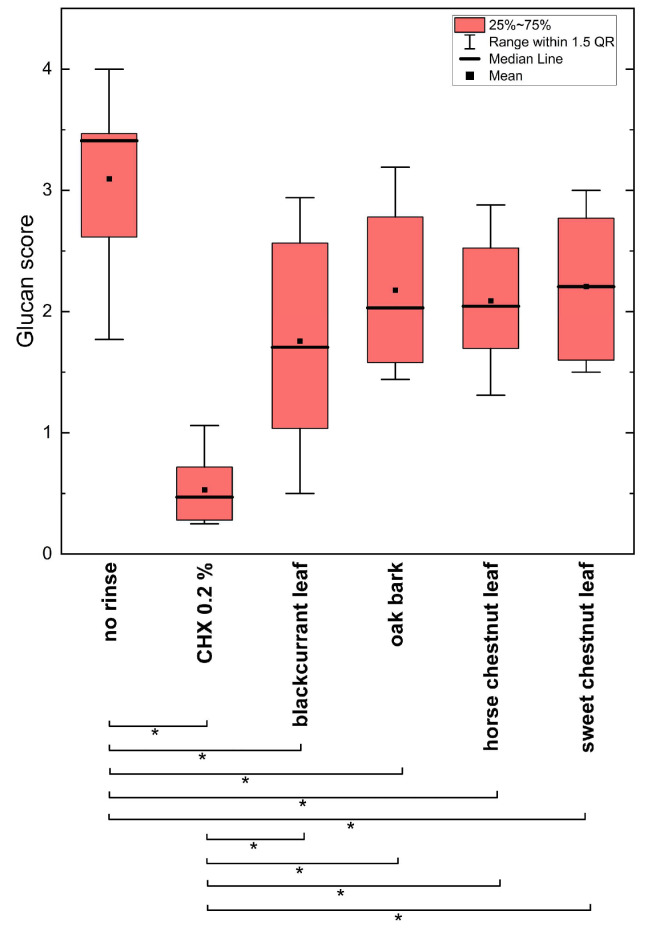
Glucan score on enamel after 8 h oral exposure following no rinse, CHX rinse, or 10 min rinsing with the four plant extracts. Asterisks indicate significant differences (Mann–Whitney U test with Bonferroni–Holm correction, *p* < 0.05).

**Figure 7:**
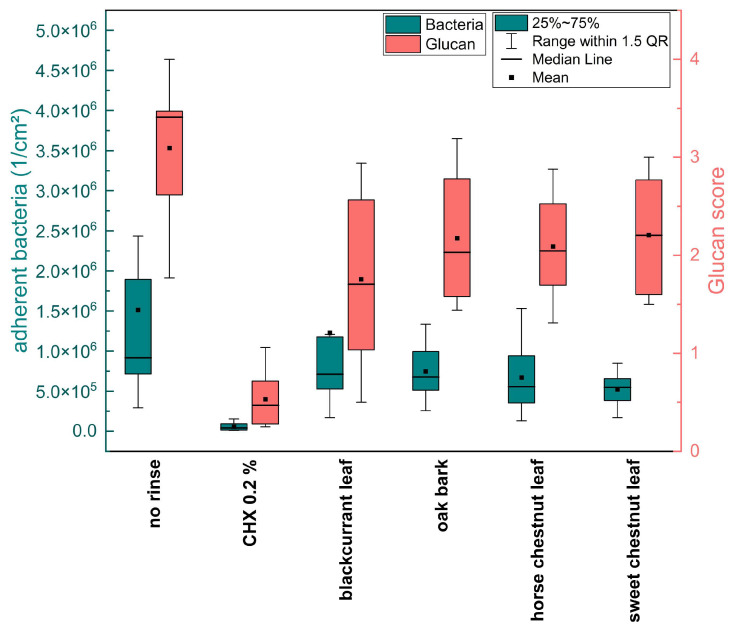
Juxtaposition of adherent bacteria and glucan scores.

Compared to the control group, no statistically significant reduction in the overall bacterial density was observed with DAPI staining after a rinse with the four screened herbal extracts, as shown in Figure 5. Similarly, no significant differences were observed among the herbal extracts in their effects on the overall bacterial density. However, the glucan score was significantly reduced after rinsing with each of the screened herbal drugs compared to the control group without a rinse (Figure 6). However, this reduction was not as extensive as that achieved by rinsing with CHX (Figure 6). Although no significant reduction in the overall bacterial density by any of the four herbal extracts was demonstrated, a significant decrease in the glucan score, used as an indicator of extracellular matrix formation, was observed (Figure 7).

**Figure 8:**
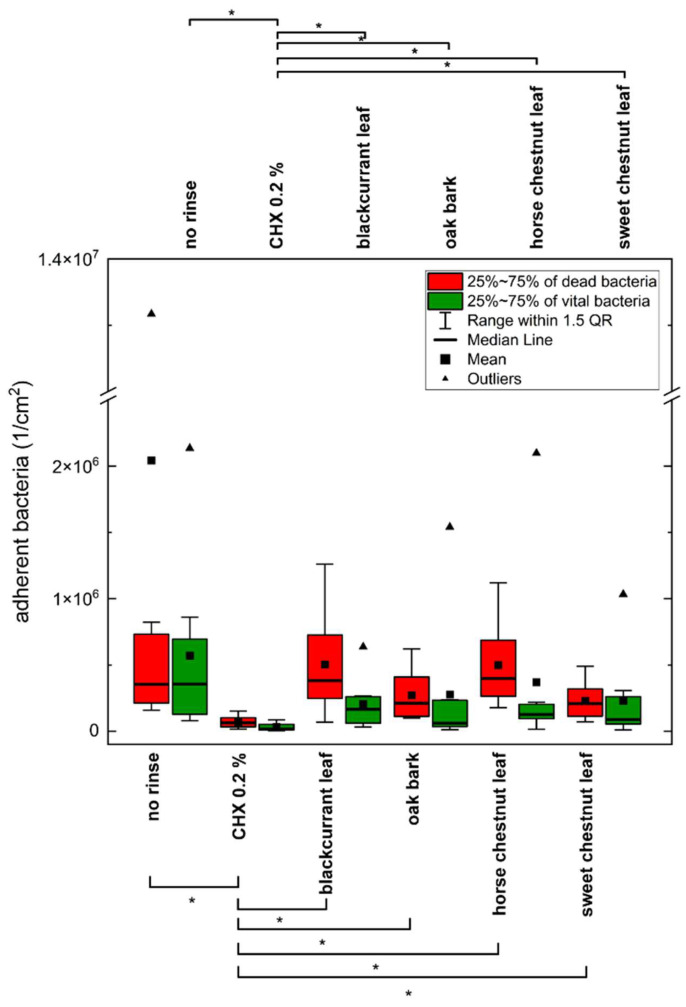
Density of adherent vital and dead bacteria on enamel after 8 h oral exposure following no rinse, CHX rinse, or 10 min rinsing with the four plant extracts. Asterisks above the figure indicate significant differences between the density of adherent vital bacteria and asteriks below the figure indicate significant differences between the density of adherent dead bacteria (Mann–Whitney U test with Bonferroni–Holm correction, *p* < 0.05).

In accordance with the DAPI staining results, LIVE/DEAD™ BacLight™ staining revealed no significant reduction by the herbal extracts in the number of dead or vital bacteria compared to the negative control. All extracts showed significantly higher levels than CHX (Figure 8).

The negative control demonstrated balanced proportions of vital and dead bacteria, with a median viability of 50%. CHX induced the strongest shift toward dead bacteria, reducing median viability to 20%. All four polyphenol-rich extracts decreased median viability relative to the negative control: blackcurrant leaves to 30%, sweet chestnut leaves to 29%, horse chestnut leaves to 24%, and oak bark to 22%. The latter approached the effect observed with CHX. Further information regarding the viability can be found in Figure S1 of the Supplementary Materials.

## 4. Discussion

In the present in situ study, no significant reduction in overall bacterial density or in the number of vital or dead adherent bacteria could be observed compared to the negative control without rinse 8 h after rinsing, suggesting that the mode of action of the investigated extracts does not directly interfere with bacterial adhesion mechanisms. Therefore, conclusions regarding the prevention of initial bacterial colonization should be interpreted with caution. However, all four extracts exhibited consistent trends toward reduced glucan formation and decreased bacterial viability (Figure 7 and Figure S1). The observed inhibition of glucan formation suggests that these plant extracts may target enzymatic pathways involved in extracellular polysaccharide synthesis. By disrupting glucan production, the structural integrity of the biofilm matrix may be compromised, which could indirectly affect bacterial survival within the biofilm environment.

In contrast to certain other polyphenolic agents, such as *Inula viscosa* and *Fragaria vesca* [39,42], no anti-adhesive effects on enamel were observed. However, shifts in bacterial colonization patterns were detected, with smaller colonies and a viability shift favoring dead cells. This was most pronounced for oak bark, which reduced bacterial viability to 22%, approaching the effect of 0.2% CHX with a viability of 20%. Further information regarding the viability can be found in Figure S1 of the Supplementary Materials.

When compared to prior in situ studies employing comparable designs, the present results align in part with published data. For example, extracts of *Fragaria vesca*, *Hamamelis*, *Tormentilla*, and tannic acid [39,43] have previously been shown to reduce the number of adherent bacteria after 8 h exposure, though without significantly lowering the proportion of vital cells. Similarly, studies on *Inula viscosa* [42] demonstrated a viability shift towards predominantly dead bacteria, consistent with the present findings. Across multiple investigations, including the current one, a consistent feature of polyphenolic plant extracts has been the inhibition of glucan formation, while anti-adhesive effects on early bacterial adhesion and biofilm development differ and appear to depend strongly on the plant source.

The pH values of the tested infusions ranged from mildly acidic (pH 5.9 for horse chestnut leaves) to more acidic (pH 4.7 for sweet chestnut leaves). Although acidic pH has been proposed to enhance polyphenol–protein aggregation and potentially facilitate stronger interactions with pellicle proteins and salivary constituents, no clear differences in efficacy were observed between the screened extracts of differing acidity [44].

Xi et al. have shown that tannic acid led to lysis of the bacterial cell wall [45]. However, in the present study no significant differences regarding initial bacterial adherence on enamel were noted between more flavonoid-rich extracts (blackcurrant leaves, horse chestnut leaves) and more tannin-rich extracts (oak bark, sweet chestnut leaves). These findings support the idea that individual polyphenolic fractions may have distinct antibacterial activities, while synergistic interactions seem to play a key role in determining overall biological effects [1,46]. The findings of this study suggest that the observed effect of reducing glucan formation may be attributed to the inhibition of enzymes directly and indirectly involved in glucan formation. Recent studies have demonstrated the capacity of polyphenols to inhibit both the bacterial isoenzymes of glycosyltransferases and the human digestive enzyme α-amylase. α-Amylase, an enzyme responsible for the hydrolysis of the 1,4-alpha-glycosidic bond, plays a pivotal role in the breakdown of starch components into smaller oligosaccharides, thereby providing the substrate for glycosyltransferases [47]. As Jeon et al. [47] have demonstrated, polyphenols that exhibit strong anti-glycosyltransferase activity are frequently condensed and/or hydrolysable tannins based on catechins or gallic acid esters. Furthermore, the inhibitory effect appears to increase with increasing polymerization of the monomeric polyphenol, which could be due to non-specific binding and precipitation of the enzymes, in the sense of a tanning effect.

Additional mechanisms proposed in the literature, such as modification of pellicle surface properties, masking of receptor proteins, disruption of microbial acidogenicity, or direct bacterial lysis, may also contribute. The absence of significant anti-adhesive effects in this study may be due to insufficient concentrations of active polyphenols, suboptimal chemical composition, or loss of activity after 8 h exposure. Since long-term effects are clinically more relevant, repeated or prolonged application regimens warrant further investigation.

The small sample size is a limitation of the study. Another limitation of the present study is the reliance on manual counting of adherent bacteria and the use of glucan scoring as a semi-quantitative assessment method. While this is a standard in comparable in situ studies [39,41,42], it is prone to observer bias and limited reproducibility. Automated programs for cell counting have difficulties differentiating between bacterial cells and other non-bacterial cells, such as desquamated epithelial cells, which were frequently found on the specimens. Individual differences in oral microbiota and daily habits may also have contributed to the lack of statistical significance in bacterial counts.

Nevertheless, the combination of the in situ model with HPLC analysis represents a valuable approach for preliminary screening potential bioactive effects under clinically relevant conditions. In the present study, this screening approach revealed only limited effects on bacterial adhesion itself, whereas effects on glucan formation and bacterial viability appeared more pronounced. In cases where stronger or more specific activities are observed, this can serve as a starting point for further mechanistic and functional investigations. Further studies involving iterative applications of the herbal drugs are possible and additional parameters and methods could be considered, such as microbiome sequencing, metabolomics, biofilm pH analysis, and dose–response investigations. Future research should extend observation periods beyond 8 h to evaluate possible cumulative effects, assess the impact on acid production by cariogenic microorganisms, and explore ecological shifts in oral microbiota following repeated application. Moreover, other herbal drugs should be screened for their potential use in oral health.

Given the growing interest in polyphenols as dietary and topical agents for oral and systemic health, their role in modifying pellicle-mediated biofilm processes remains an attractive field of investigation. Particularly promising are combination strategies, pairing polyphenolic extracts with fluorides or stannous ions to achieve complementary mechanisms of action, especially anti-erosive effects [48]. Flemming at al. found that SnCl2 and SnF2, as well as their combinations with tannic acid, led to a reduction in initial bacterial colonization and glucan formation and show an erosion-protective effect [41].

## 5. Conclusions

The present findings suggest that polyphenol-rich plant preparations may differ in their effects on early bacterial adhesion and biofilm development. While none of the four extracts showed a significant reduction in the overall number of adherent bacteria, all four showed a significantly reduced glucan formation and a trend toward a decreased bacterial viability in situ. These effects suggest that certain polyphenol-containing preparations may influence specific early biofilm processes, although their effectiveness appears to depend strongly on the plant source. Due to the heterogenous nature of polyphenols and the diversity of bacteria in dental biofilms, it is not possible to draw a generalized conclusion about the effect of polyphenolic teas on dental biofilm formation. Because this study was designed as a screening approach, the results should be interpreted as an initial assessment rather than evidence of efficacy. In contrast to previously investigated extracts such as *Fragaria vesca*, *Hamamelis virginiana*, and *Tormentil* [39] or tannic acid [4], the tested herbal preparations did not yield comparable effects on initial bacterial colonization. Nevertheless, the consistent tendencies observed for glucan reduction and impaired bacterial viability justify further investigation. Within the limits of this in situ model supported by detailed chemical analysis, the tested extracts can be considered preliminary candidates for follow-up research. Future studies should examine additional plant species and combinations, and assess their effects under repeated or longer-term exposure and in more complex oral environments. This will help clarify which herbal drugs may be integrated into a health-promoting diet or adjuvant oral care products, contributing to a sustainable improvement of both oral and general health.

## Figures and Tables

**Table 1:** Herbal drugs used in this study.

Blackcurrant leaves	*Ribes nigrum*, leaves of *Ribes nigrum* L., cut Batch no.: 79927, Kräuter Schulte, Gernsbach/Schwarzwald, Germany
Sweet chestnut leaves	*Castaneae folium*, leaves of *Castanea sativa* Mill., cut Batch no.: 83043, Kräuter Schulte, Gernsbach/Schwarzwald, Germany
Horse chestnut leaves	*Hippocastani folium*, leaves of *Aesculus hippocastanum* L., cut Batch no.: 84654, Kräuter Schulte, Gernsbach/Schwarzwald, Germany
Oak bark	*Quercus* cortex, bark from young branches of *Quercus robur* L. or *Quercus petraea* Liebl. Batch no.: 83042, Kräuter Schulte, Gernsbach/Schwarzwald, Germany

**Table 2:** Evaluation scheme for the extent of glucan formation.

Glucan Score	Evaluation
0	No recognizable glucan rings
1	Individual glucan rings or bundles
2	Some distinct rings are visible, while there are also less prominent rings and clusters around the bacteria
3	At least 50% of bacteria have distinct glucan rings
4	Clear glucan rings in (almost) all bacteria

**Table 3:** pH values of the extracts.

Extracts	pH Value
horse chestnut leaves	5.93
blackcurrant leaves	5.80
oak bark	4.93
sweet chestnut leaves	4.70

## Data Availability

The data that support the findings of this study are available from the corresponding author on reasonable request.

## Supplementary Materials

The following supporting information can be downloaded at: https://www.mdpi.com/article/10.3390/plants15132101/s1, Figure S1: Bacterial density by vitality status and median proportions of vital and dead cells; Supplementary Text S1: Detailed characterization of the individual phenolic constituents and their quantitative data for the four investigated herbal drugs (blackcurrant leaves, horse chestnut leaves, sweet chestnut leaves, and oak bark).

## Abbreviations

CHX	Chlorhexidine gluconate
h	Hours
min	Minutes
sec	Seconds
DAPI	4′,6-diamidino-2-phenylindole
Con A	Concanavalin A
