# Supplementary material for: Composition of Different Herbal Extracts and Their Impact on Initial Bacterial Colonization on Enamel In Situ
Source: Plants (Basel). 2026 Jul 7;15(13):2101. doi: 10.3390/plants15132101 (PMC13364459; doi:10.3390/plants15132101)
Supplement: Supplementary file 1 [file plants-15-02101-s001.zip › plants-4322590-supplementary.pdf]

Supplementary information

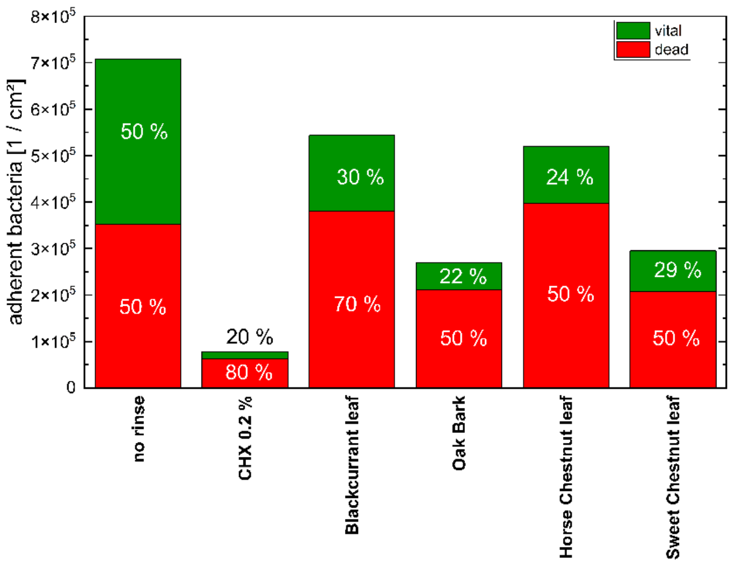

Figure S1 Bacterial density by vitality status and median proportions of vital/dead cells

Detailed characterization of the individual phenolic constituents and their quantitative data

S1.1 Blackcurrant Leaves

In the blackcurrant leaves, three groups of polyphenols were detected: phenolic acids, flavonols, and tannins. For the first group, quinic acid, gallic acid (10 mg/100 g; G), protocatechuic acid, p-hydroxybenzoic acid, p-coumaric acid (70 mg/100 g; C), caffeic acid (47 mg/100 g; C), and 3-chlorogenic acid (3-CQA) (18 mg/100 g; C), 4-CQA (13 mg/100 g; C), 5-CQA (119 mg/100 g; C), 5-coumaroyl quinic acid (13 mg/100 g; C), and a caffeoyl-glucoside (19 mg/100 g; C) were identified [1].

Within the group of flavonols glycosides of myricetin, quercetin, kaempferol, and isorhamnetin were identified. Myricetin-3-O-rutinosid and -glucosid (21 mg/100 g; R) as well as myricetin-malonyl-glucoside were detected in the leaves of blackcurrants, in addition to quercetin-3-O-rutinosid (141 mg/100 g; R), -glucosid (267 mg/100 g; R), and quercetin-malonyl-glucoside (317 mg/100 g; R). Furthermore, kaempferol-3-O-rutinosid (48 mg/100 g; R), -glucosid (107 mg/100 g; R) and a kaempferol-malonyl-glucoside (119 mg/100 g; R) were

identified. The isorhamnetin glycoside was determined as isorhamnetin-3-O-rutinosid [1,2]. The three malonyl-glucosides were identified via an extract of apocynum venetum [2].Except of two isomers of monogalloylhexose only condensed tannins were detected. The identified monomers gallocatechin (13 mg/100 g; G) and epigallocatechin are precursors of prodelphinidines. One prodelphinidin dimer und one trimer could be identified (7 and 10 mg/100 g; G). Prodelphinidine in the leaves of blackcurrants were also reported by Liu et al. [3]. The monomer (+)-catechin (40 mg/100 g; G) was described by Böhme [1].

S1.2 Horse Chestnut Leaves

In the leaves of the horse chestnut, besides the phenolic acids, flavonols, and condensed tannins a fourth group was determined with the cumarin derivatives. As phenolic acids quinic acid, protocatechuic acid (13 mg/100 g; G), coumaric acid (12 mg/100 g; C), and 3-CQA (21 mg/100 g; C) were described in horse chestnut leaves for the first time, caffeoyl-hexose-rhamnose (16 mg/100 g, C) was confirmed [4]. In the group of the flavonols the quercetin glycosides rutin (16 mg/100 g; R), quercetin-3-O-rhamnosid (412 mg/100 g; R), -3-O-arabinosid (136 mg/100 g; R), -3-O-glucosid (33 mg/100 g; R), and -3-O-galactosid (24 mg/100 g; R) were analyzed as components. Furthermore, a quercetin-triglycoside and a quercetin-dirhamnoside (18 mg/100 g; R) were suspected due to their mass fragmentation behaviour. Kaempferol-3-O-arabinoside (15 mg/100 g; R) and kaempferol-3-O-rhamnoside (60 mg/100 g; R) could be confirmed [4]

In the tannin group besides the monomer (-)-epicatechin (22 mg/100 g; G) several procyanidines of the A- and of the B-typs were identified. Epicatechin-(2β→7,4β→8)-epicatechin as the procyanidin-A2-dimere (27 mg/100 g; G) was elucidated by a standard substance. In addition, two procyanidin-A-trimeres (23 mg/100 g; G) and two procyanidin-A-tetrameres (9 and 6 mg/100 g; G) were detected [4]. The procyanidin-B2-dimere (9 mg/100 g) was quantified via a standard substance.

Regarding the coumarin derivatives, esculetin (23 mg/100 g; C) and fraxetin (8 mg/100 g; C) both substances were identified in the leaves for the first time, moreover the expected glucosides esculin (61 mg/100 g; C) and fraxin (20 mg/100 g; C) could be determined by using standard substances.

S1.3 Sweet Chestnut Leaves

The leaves of the sweet chestnut contained phenolic acids, flavonols, and despite to the hitherto described matrices with ellagitannins numerous hydrolysable tannins.

The phenolic acids gallic acid (179 mg/100 g; G), ellagic acid (155 mg/100 g; EA), and 5-coumaroyl quinic acid (30 mg/100 g; C), and also quinic acid and 5-CQA (29 mg/100 g; C), were described for the first time. The components quercetin-3-O-rutinoside (41 mg/100 g; R), -galactoside (29 mg/100 g; R) und -glucoside (219 mg/100 g; R), as well as quercetin-glucuronide (232 mg/100 g; R) have already been mentioned in the literature [5,6] In addition, quercetin-3-(p-coumaryl)-hexoside (21 mg/100 g; R), and a quercetin-pentoside (10 mg/100 g; R) could be assigned due to mass spectrometric data. The flavonolglycosides kaempferol-3-O-rutinoside (21 mg/100 g; R), and -glucoside (115 mg/100 g; R), as well as isorhamnetin-3-O-rutinoside (15 mg/100 g; R) could be confirmed [5,6]. The isorhamnetin-glucuronide (13 mg/100 g; R) and the isorhamnetin-3-O-glucoside (31 mg/100 g; R) were identified in the leaves for the first time [7]. Moreover, an isorhamnetin-pentoside (3 mg/100 g; R) was detected. Kaempferol-3-(6''-p-coumaryl)-glucoside (Tilirosid) (3 mg/100 g; C) was at first proposed by Romussi et al. [8] for fresh leaves using IR-spectroscopy and now verified by a standard substance. In addition, a kaempferol-acetyl-hexoside (3 mg/100 g; R), and a kaempferol-dicoumaryl-hexoside (13 mg/100 g; C) were identified on the basis of mass spectroscopic data. The latter substance was already described by Esposito et al. [7] in the burs of the sweet chestnut.

The ellagitannins vescalagin (374 mg/100 g; V) and castalagin (132 mg/100 g; V) were identified. Vescavalonic- and castavalonic acid first described by Esposito et al. [7] to be part of the burs of the sweet chesnut, were now determined in the leaves for the first time (135/92 mg/100 g; V). Furthermore, the ellagitannines pedunculagin isomere 1 und 2 (101/102 mg/100 g; V), chesnatin isomere 1 bis 3 (393/257/53 mg/100 g; G), casuarinin (98 mg/100 g; G) und cocciferin D2 (246 mg/100 mg; V) were detected. Casuarinin was confirmed by analyzing a pomegranate extract [9]. Based on an oak bark extract as reference castalin, grandinin und roburin E were identified [10]. Grandinin und roburin E were only detected in the bark of the sweet chestnut so far [11]. With the proof of chestanin the findings by Cerulli et al. [12] were confirmed, and in addition a second chestanin isomere was detected (185/12 mg/100 g; G). Furthermore, tellimagrandin I (73 mg/100 g; V) and potentillin were identified using mass spectroscopy. Both substances have been identified by paper chromatography so far [13].

S1.4 Oak bark

In the oak bark sample, phenolic acids and in particular flavonols were only detected as minor compounds, in contrary to ellagic acid, condensed, hydrolysable and complex flavotannins. With quinic acid and gallic acid (38 mg/100 g; G), only two phenolic acids were examined. With the procyanidin-dimer B3, proofed via standard substance (31 mg/100 g; G), and a procyanidin dimere (45 mg/100 g; G), two condensed tannins were identified.

Ellagic acid (18 mg/100 g; EA), an ellagic acid pentoside, ellagic acid rhamnoside (20 mg/100 g; R) as well as an ellagic acid hexoside, and methylellagic acid were detected (Fernandes et al., 2011). Ellagitannins are the dominant components in oak bark. The isomers vescalagin (8 mg/100 g; EA) and castalagin (37 mg/100 g; EA) were proofed via standard substances, castalin (4 mg/100 g; EA), roburin E or grandinin, has been expected and were confirmed.

With acutissimin (37 mg/100 g; EA), a flavanol ellagitannin composed of vescalagin and catechin/epicatechin, and its oxidation product mongolicain (13 mg/100 g; EA), two complex tannins were assigned. Three major peaks could not clearly identified, two of them were possibly monogalloyl dihexoside and methylellagic acid rhamnoside.

1. Böhme, A. Analyse Und Antimikrobielle Untersuchung Sekundärer Inhaltsstoffe in Den Blättern Der Schwarzen Johannisbeere, TU Dresden, 2013.
2. Hauke-Paskuy, C. Flavonolglykoside in Den Blättern Der Schwarzen Johannisbeere Und Anderer Ribes-Arten, TU Dresden, 2015.
3. Liu, P.; Kallio, H.; Yang, B. Flavonol Glycosides and Other Phenolic Compounds in Buds and Leaves of Different Varieties of Black Currant (Ribes Nigrum L.) and Changes during Growing Season. *Food Chem.* **2014**, *160*, 180–189, doi:10.1016/j.foodchem.2014.03.056.
4. Oszmiański, J.; Kalisz, S.; Aneta, W. The Content of Phenolic Compounds in Leaf Tissues of White (Aesculus Hippocastanum L.) and Red Horse Chestnut (Aesculus Carea H.) Colonized by the Horse Chestnut Leaf Miner (Cameraria Ohridella Deschka & Dimić). *Molecules* **2014**, *19*, 14625–14636, doi:10.3390/MOLECULES190914625.
5. Silva, V.; Falco, V.; Dias, M.I.; Barros, L.; Silva, A.; Capita, R.; Alonso-Calleja, C.; Amaral, J.S.; Igrejas, G.; Ferreira, I.C.F.R.; et al. Evaluation of the Phenolic Profile of Castanea Sativa Mill. By-Products and Their Antioxidant and Antimicrobial Activity against Multiresistant Bacteria. *Antioxidants 2020, Vol. 9, Page 87* **2020**, *9*, 87, doi:10.3390/ANTIOX9010087.
6. Munekata, P.E.S.; Franco, D.; Trindade, M.A.; Lorenzo, J.M. Characterization of Phenolic Composition in Chestnut Leaves and Beer Residue by LC-DAD-ESI-MS. *LWT - Food Sci. Technol.* **2016**, *68*, 52–58, doi:10.1016/J.LWT.2015.11.017.
7. Esposito, T.; Celano, R.; Pane, C.; Piccinelli, A.L.; Sansone, F.; Picerno, P.; Zaccardelli, M.; Aquino, R.P.; Mencherini, T. Chestnut (Castanea Sativa Miller.) Burs Extracts and Functional Compounds: UHPLC-UV-HRMS Profiling, Antioxidant Activity, and Inhibitory Effects on Phytopathogenic Fungi. *Mol. 2019, Vol. 24, Page 302* **2019**, *24*, 302, doi:10.3390/MOLECULES24020302.
8. Romussi, G.; Mosti, L.; Bignardi, G. Inhaltsstoffe von Cupuliferae, II Ein Neues Acyliertes Flavonoidglykosid Aus Castanea Sativa Mill. *Liebigs Ann. der Chemie* **1981**, *1981*, 761–764, doi:10.1002/JLAC.198119810502;JOURNAL:JOURNAL:10990690C;PAGE:STRING:ARTICLE/C

HAPTER.
9. Seeram, N.P.; Schulman, R.N.; Heber, D. *Pomegranates: Ancient Roots to Modern Medicine.*; CRC Press, 2006; ISBN 9780429125539.
10. Du Penhoat, C.L.M.H.; Michon, V.M.F.; Peng, S.; Viriot, C.; Scalbert, A.; Gage, D. Structural Elucidation of New Dimeric Ellagitannins from Quercus Robur L. Roburins A–E. *J. Chem. Soc., Perkin Trans. 1* **1991**, 1653–1660, doi:10.1039/P19910001653.
11. Comandini, P.; Lerma-García, M.J.; Simó-Alfonso, E.F.; Toschi, T.G. Tannin Analysis of Chestnut Bark Samples (Castanea Sativa Mill.) by HPLC-DAD–MS. *Food Chem.* **2014**, *157*, 290–295, doi:10.1016/J.FOODCHEM.2014.02.003.
12. Cerulli, A.; Masullo, M.; Mari, A.; Balato, A.; Filosa, R.; Lembo, S.; Napolitano, A.; Piacente, S. Phenolics from Castanea Sativa Leaves and Their Effects on UVB-Induced Damage. *Nat. Prod. Res.* **2018**, *32*, 1170–1175, doi:10.1080/14786419.2017.1331225.
13. Hänsel, R..; Keller, K..; Rimpler, H..; Schneider, G..; Aye, R.D..; Bauer, I..; Baumann, T.W..; Beck, M..; Beil, A..; Blascheck, W..; et al. *Drogen A-D*; Springer Berlin Heidelberg : Imprint : Springer, 1992; ISBN 978-3-642-63468-0.
